# Critical assessment of pan-genomic analysis of metagenome-assembled genomes

**DOI:** 10.1093/bib/bbac413

**Published:** 2022-09-17

**Authors:** Tang Li, Yanbin Yin

**Affiliations:** Nebraska Food for Health Center, Department of Food Science and Technology, University of Nebraska - Lincoln, Lincoln, NE, 68508, USA; Nebraska Food for Health Center, Department of Food Science and Technology, University of Nebraska - Lincoln, Lincoln, NE, 68508, USA

**Keywords:** metagenome, microbiome, MAG, metagenome-assembled genomes, pan-genome, pan-genomics

## Abstract

Pan-genome analyses of metagenome-assembled genomes (MAGs) may suffer from the known issues with MAGs: fragmentation, incompleteness and contamination. Here, we conducted a critical assessment of pan-genomics of MAGs, by comparing pan-genome analysis results of complete bacterial genomes and simulated MAGs. We found that incompleteness led to significant core gene (CG) loss. The CG loss remained when using different pan-genome analysis tools (Roary, BPGA, Anvi’o) and when using a mixture of MAGs and complete genomes. Contamination had little effect on core genome size (except for Roary due to in its gene clustering issue) but had major influence on accessory genomes. Importantly, the CG loss was partially alleviated by lowering the CG threshold and using gene prediction algorithms that consider fragmented genes, but to a less degree when incompleteness was higher than 5%. The CG loss also led to incorrect pan-genome functional predictions and inaccurate phylogenetic trees. Our main findings were supported by a study of real MAG-isolate genome data. We conclude that lowering CG threshold and predicting genes in metagenome mode (as Anvi’o does with Prodigal) are necessary in pan-genome analysis of MAGs. Development of new pan-genome analysis tools specifically for MAGs are needed in future studies.

## Introduction

The term pan-genome was first proposed in bacteria in 2005, representing the entire gene set of all strains in a species [1]. Genes in a pan-genome are classified into three categories: core, accessory and unique. In theory, core genes (CG) must be shared by all strains within the species, accessory genes are present in a subset of the strains, and unique genes are only present in a specific strain [1, 2]. The strictest CG threshold is 100%, meaning that CGs are present in 100% of genomes. However, in practice, CGs can be defined with a more relaxed threshold (e.g. 95%). Since 2005, pan-genome analysis has become an essential component of comparative genomics study not only in prokaryotes but also in plants [3], fungi [4], animals [5] and humans [6]. In bacteria, pan-genome analysis has broad applications in studying genomic diversity and phylogeny [7, 8], disease outbreak [9], virulence-associated genes [10] and antimicrobial resistance [2, 11].

A great number of computational tools for bacterial pan-genome analysis have been developed, such as PGAP [12], GET_HOMOLOGUES [13], ITEP [14], Roary [15], Anvi’o [16], BPGA [17] and PanX [18]. Surveys and comparisons of these tools have been published in recent years [2, 11, 19, 20]. One recent study [21] revealed that the incomplete and inconsistent gene annotations may lead to underestimated core genome size and overestimated pan-genome size. This is particularly important as it is now very common to include draft isolate genomes and metagenome-assembled genomes (MAGs) in pan-genome analysis.

MAGs are produced from metagenome shotgun sequencing reads through filtering, assembling, binning and taxonomy assignment to generate the approximate representation of actual individual genomes. The term ‘MAG’ first appeared in the literature in 2015 [22, 23], when the first large-scale MAG study was published [24]. In 2017, the Genomic Standards Consortium (GSC) published the Minimum Information about a MAG (MIMAG), a metagenomics community standard for releasing MAGs with mandatory metrics (genome completeness and contamination) [25]. Since then, hundreds of thousands of MAGs have been reconstructed from various environments, e.g. ocean [26], soil [27], freshwater [28], human gut [29, 30], activated sludge [31] and animal gut [32, 33]. These MAGs are extremely useful to improve predictions of metabolic capacities, discover completely novel taxa, and expand the tree of life [34, 35]. However, a recent study revealed that MAGs with 95% completeness captured only ~77% population CGs and ~50% variable genes, and the quality of MAGs was often worse than expected [36]. Another study [35] critically reviewed concerns that would significantly limit the use of MAGs, such as gaps, assembly and binning errors, chimeras, and contaminations. Even high-quality MAGs (>90% completeness and <5% contamination according to MIMAG) may still contain assembly errors and chimeras [25, 37].

Since 2017, MAGs are increasingly used in pan-genome analyses (see Supplementary Figure S1, Supplementary Table S1, Supplementary Results). For example, studies have been published using MAGs in pan-genome analysis of human microbiomes [30, 38–41], hydrothermal vents [42, 43], ocean [44] and animal gut [45]. Obviously, it has become a routine to combine MAGs with isolate genomes or use only MAGs for pan-genome analysis. However, to which extent the accuracy of pan-genome results may be influenced by the caveats (fragmentation, incompleteness and contamination) of MAGs has never been critically assessed. We hypothesize that including MAGs will lead to biases and errors in pan-genome analysis results and further influence the downstream functional and phylogenetic analysis. To test this hypothesis, we have compared pan-genome analysis results of complete genomes and MAGs simulated from complete genomes by introducing fragmentation, incompleteness and contamination. Based on our findings, we have provided recommendations to alleviate the accuracy loss due to the use of MAGs: (1) choose more relaxed CG thresholds, e.g. 90% or 95%, (2) select gene prediction tools considering fragmented genes, (3) use mixed datasets including MAGs and complete genomes and (4) perform pan-genome analysis using newer tools designed for low-quality isolate genomes or MAGs.

## Materials and methods

### Simulate MAGs from complete genomes

**Figure 1:**
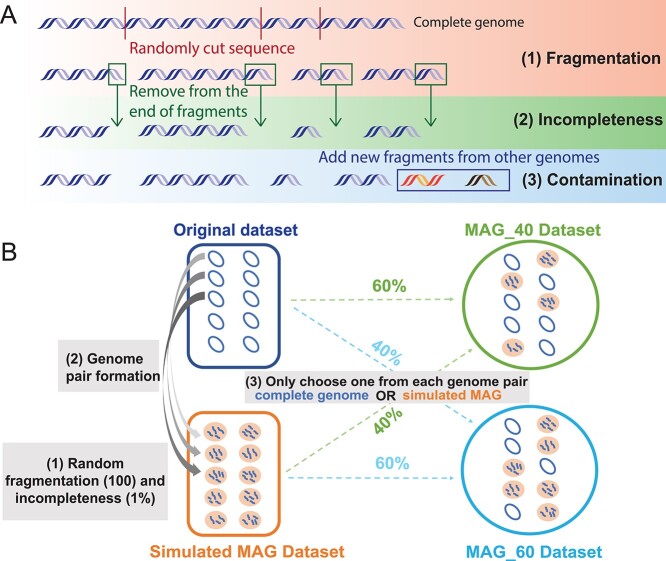
Workflows to generate simulated MAG datasets. (**A**) The pipeline to generate fragmented, incomplete and contaminated MAGs from complete genomes. The first step fragmentation can be performed at five different levels (50, 100, 200, 300 and 400 fragments). The second step incompleteness can be performed at five different levels (1, 2, 3, 4 and 5%). The third step contamination can be performed at eight different levels (0.5, 1, 1.5, 2, 2.5, 3, 3.5, 4%). (**B**) Combining complete genomes and simulated MAGs to form mixed datasets. Shown in the diagram as an example, the original dataset contains 100 randomly selected complete genomes. Genomes in the simulated MAG dataset are generated from complete genomes by fragmentation and incompleteness simulation (described in A). Each genome pair contains one complete genome and its corresponding MAG. Two mixed datasets are shown as examples. MAG_40 is created by randomly combining complete genomes (60%) in the original dataset and MAGs (40%) from the simulated MAG dataset. If a complete genome has been chosen, its corresponding MAG would not be selected.

To simulate MAGs from complete genomes, all complete genomes of 17 bacterial species were downloaded from the NCBI RefSeq database in October 2019 [46]. Each species has at least 100 complete genomes in the database (Supplementary Figure S2, Supplementary Table S2). For each species, 100 complete genomes were randomly selected as the ‘*original dataset.’* MAGs were simulated from the complete genomes resembling the distribution of fragmentation, completeness, and contamination observed in Unified Human Gastrointestinal Genome (UHGG) MAGs [38]. Fragments from genomes of the same species or genus were added as contamination. The simulation process was depicted in Figure 1A, the details was described in Supplementary Methods. Overall, four types of datasets were generated: (1) *original datasets* (100 complete genomes), (2) *fragmentation datasets* (fragmented MAGs), (3) *incompleteness datasets* (fragmented + incomplete MAGs) and (4) *contamination datasets* (fragmented + incomplete + contaminated MAGs). These simulated MAG datasets (*fragmentation, incompleteness* and *contamination*) were used to compare with original datasets in the next steps.

### Mixed and real MAG datasets

MAGs are often combined with complete isolate genomes together for pan-genome analysis (Supplementary Figure S1, Supplementary Table S1). Therefore, we have created mixed datasets for two representative species, *Escherichia coli* and *Bordetella pertussis*, to compare with the original datasets. Four different MAG percentages were used: 10%, 40%, 60% and 85%. For example, to create a mixed dataset with 40% MAGs, a total of 40 MAGs (40% in 100) would be randomly selected from the simulated MAG dataset (Figure 1B).

In addition, the real MAG datasets contain five pathogenic *E. coli* MAGs and their corresponding isolate genomes from Meziti *et al.* [36]. They were used as the real datasets to test our findings made in simulated data. Details can be found in Supplementary Methods.

### Pan-genome analyses

Once the input datasets (original genomes, simulated MAGs or mixed MAGs) were built, pan-genomes were constructed for these datasets and compared. Proteins were predicted and annotated using Prokka v1.13 [47] with default parameters for all genomes/MAGs. Pan-genome analyses were carried out using tools such as Roary v3.13 [15]. Simulated MAGs were generated at different levels of fragmentation, incompleteness and contamination (Figure 1). For example, five different levels (50, 100, 200, 300 and 400) of fragmented MAGs were subject to pan-genome analysis. To project the correlation between the number of CG families and the number of fragments, an exponential model ($y={e}^{\big( ax+b\big)}$) was used, where *y* is the number of the CG families in pan-genome and *x* represents the number of fragments. The fitting curves were predicted with adjusted-*R*^2^ and *P*-values.

### Test the effect of random selection of complete genomes

To assess the variation that may be caused by the selection of different 100 genomes for simulation, multiple datasets were used to repeat pan-genome analyses. 50 random datasets (each with 100 randomly selected genomes) were generated for *E. coli* by going through the same simulation process as depicted in Figure 1. For *B. pertussis, Staphylococcus aureus,* and *Klebsiella pneumoniae*, 30 random datasets were generated (these species have smaller numbers of complete genomes). The datasets of the same type (e.g. 100cut fragmentation group) were analyzed separately. The median, mean and standard deviation for the number of CGs of the 50 or 30 datasets were calculated.

### Compare three pan-genome analysis tools

Three pan-genome analysis tools, Roary [15], BPGA v1.3 [17] and Anvi’o v7.0 [16], were compared for two representative species, *E. coli* and *B. pertussis*. To have a fair comparison, all three tools used the same gene prediction files from Prokka v1.13 [47], unless stated differently.

Roary was run with the parameters ‘-i 90 -cd 100 -s -e -n’, where -i defines the sequence identity (SI) threshold in BLAST comparison, and -cd defines the percentage threshold in CG definition (see below). These two parameters can be set at different thresholds. In BPGA, USEARCH [48] was selected as the gene clustering tool with the same SI and CG thresholds used in Roary. In Anvi’o, the internal gene prediction was skipped, and a python script was used to feed in the external gene prediction file (Prokka result). There is no SI parameter in Anvi’o, so two equivalent ‘--mcl-inflation 10 --use-ncbi-blast --minbit 0.8’ were used instead.

### Test the use of different SI and CG thresholds in pan-genome analysis

In pan-genome analysis, two parameters are very important: (i) the sequence identity (SI) to define homology (e.g. two genes must be >90% identical to be clustered into the same homologous gene cluster) and (ii) the CG definition threshold (e.g. CG threshold of 100% means CGs must be found in 100% of genomes, and CG threshold of 90% means CGs must be found in > = 90% of genomes). The simulated MAGs for *E. coli* and *B. pertussis* were used to evaluate the effects of different parameter thresholds on pan-genome analysis results. Different CG thresholds were compared using Roary, BPGA, and Anvi’o (use the default meta-prodigal option): 100%, 99%, 98%, 95%, 92% and 90%. Different SI thresholds were tested using Roary only: 95%, 90%, 85% and 80%.

Lowering the CG threshold will increase the CG number, but the recovered CGs may not be the lost ones due to using MAGs. To test this, the protein sequences of representative CGs in a simulated MAG dataset with a lower CG threshold (e.g. 90%) were searched against the representative CGs in the original dataset by using BLASTp to determine the true positive and false positive rate. In addition, lowering the CG threshold when using complete genomes will also lead to an increased CG number. The increased CG coverage was calculated as the number of increased CGs shared in simulated (e.g. at 90% CG threshold) and original datasets divided by the total number of increased CGs in the original dataset (see Supplementary Figure S9 for a graphical illustration).

### Downstream analysis

Two downstream analyses were performed for *E. coli* and *B. pertussis* datasets based on the Roary pan-genome results to evaluate the effects caused by MAG pan-genome: (i) clusters of orthologous genes (COG) functional analysis. The representative protein sequences of three gene groups (core, accessory and unique) were searched against the COG database [49] using RPS-BLAST with *E*-value < 1E-5. The COG functional enrichment was tested by using the one-tailed Binomial test with adjusted *P*-value >0.05 following our previous papers [50]. (ii) Phylogenetic tree comparison. The single-copy CG alignment was used to construct phylogeny by using Fasttree v2.1 [51] and RAxML [52]. The phylogeny was also reconstructed using the presence and absence of accessory genes. To compare two phylogenies and quantify the differences, the normalized Robinson-Foulds (nRF) symmetric distance [53] and the generalized RF (tree distance) [54] between the tree from the original complete genomes and the tree from simulated MAGs were calculated by using ETE3 toolkit [55] and TreeDist R package [54].

## Results

### Overview of the data and analytic approaches

Based on the literature search result (Supplementary Table S1, Supplementary Figure S1, Supplementary Results), we decided to use: (1) three popular pan-genome analysis tools (Anvi’o, Roary and BPGA), (2) the strictest CG definition threshold 100% (meaning CGs must be found in 100% of the analyzed genomes/MAGs) and (3) the dataset size of 100 genomes/MAGs for our pan-genome benchmarking. We have generated MAGs from complete bacterial genomes by simulating MAG characteristics (Figure 1A) based on the statistics of real MAGs from UHGG (Unified Human Gastrointestinal Genome) [38] (Supplementary Results). We have also repeated all the analyses on a real data that contain 5 *E. coli* isolate-MAG pairs from human fecal samples.

In this study, a total of 17 bacterial species were selected for simulation and evaluation (Supplementary Table S2). *E. coli* and *B. pertussis* were selected as representative species according to their very different characteristics (Supplementary Results, Supplementary Figure S2). The pan-genomes of the simulated MAG datasets (Figure 1, Supplementary Methods) and complete genome datasets (*original datasets*) were compared to: (1) determine the effects on core genome size caused by MAG characteristics, (2) reveal the CG loss when using different tools and datasets, (3) evaluate the CG threshold selection and (4) estimate the influences on the downstream functional and phylogenetic analysis.

### Core gene loss caused by MAG characteristics

Complete genomes usually consist of one or two chromosomes, and sometimes a few plasmids. In comparison, MAGs reconstructed from metagenome samples are highly fragmented, rarely complete, and possible contaminated (e.g. see Supplementary Figure S3 for UHGG data). In this section, the pan-genomes of simulated MAG datasets and original datasets (complete genomes) are compared to study the effects caused by fragmentation, incompleteness, and contamination. In this section, we used only Roary, which was run with 90% sequence identity (SI) and 100% CG definition thresholds.

**Figure 2:**
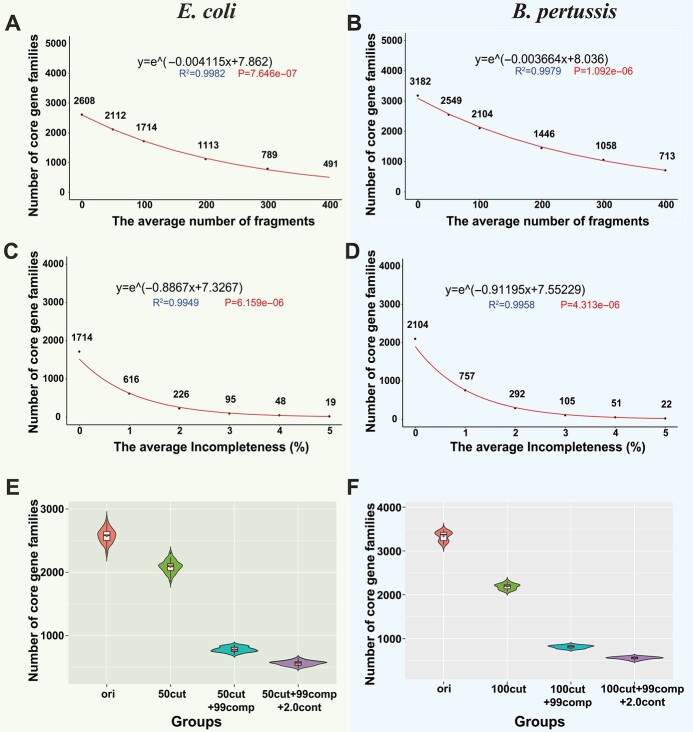
Core genome sizes decrease in simulated MAGs of *Escherichia coli* and *Bordetella pertussis*. (**A**) and (**B**) The core genome size continuously decreases as the simulated MAGs become more fragmented. The red curve was predicted using an exponential model for the correlation between the *x*-axis (the number of fragments) and the *y*-axis (the core genome size). (**C**) and (**D**) The core genome size decreases more rapidly as the simulated MAGs become less complete. (**E**) The violin plot of the core genome sizes in 50 *E. coli* original datasets and their corresponding simulated MAG datasets. (**F**) The violin plot of the core genome sizes in 30 *B. pertussis* original datasets and their corresponding simulated MAG datasets. Groups: ‘ori’ represents the original datasets; ‘50cut’ or ‘100cut’ represents the fragmentation datasets; ‘50cut + 99comp’ or ‘100cut + 99comp’ means that genomes in a dataset have an average of 1% incompleteness based on 50 or 100 fragmentation; ‘50cut + 99comp + 2.0cont’ or ‘100cut + 99comp + 2.0cont’ means that genomes in a dataset have an average of 2.0% intra-species contamination based on 50 or 100 fragmentation and 1% incompleteness. All the core genome sizes are calculated by using Roary with 90% identity and 100% CG thresholds.

As expected, the number of CG families decreased with more fragmented MAGs in *E. coli* (Figure 2A) and *B. pertussis* (Figure 2B). The reduction of core genome sizes was also observed in other 15 species (Supplementary Figure S4A). To quantitatively evaluate the degree of CG loss in different species, an exponential model was used (see Methods and Figure 2). In general, species with a larger number of CG families in the original genomes tend to have more rapid core genome reduction. Additionally, the CG loss caused by fragmentation was observed, regardless of using the average number of fragments (e.g. fragmentize each genome into 50 contigs as a simulated MAG, Figure 2, Supplementary Figure S4A, Supplementary Methods) or the average fragment length (e.g. fragmentize each genome into contigs with an average length = 100 kb a simulated MAG, Supplementary Figure S5) for the simulations.

Compared to fragmentation, incompleteness (incomplete MAGs with a fixed fragmentation rate: average 100 contigs in each MAG) had more significant effects on the core genome size. Since some genome sequences were removed in the incompleteness step, more genes would be lost in this step compared to that only cutting the genome to fragments. Only 36% (616/1714 in *E. coli* and 757/2104 in *B. pertussis*) of CG families were retained in these two species when an average of 1% genome sequences was removed (Figure 2C and D). Similar results were observed in all other species regardless of their original core genome sizes (Supplementary Figure S4B). Overall, a 1% loss in completeness, from 100% to 99%, would lead to >60% loss of CGs in most species. A 5% loss in genome completeness (Figure 2C and D, Supplementary Figure S4B) would almost lose all CGs.

Real MAGs are built by binning metagenome contigs based on similar sequence compositions and sequencing coverage [35]. Therefore, contamination is most likely from closely related genomes as they share more similar sequence compositions, which is another main difference between MAGs and complete isolate genomes. As mentioned in Methods and Supplementary Methods, we simulated contaminated MAGs by adding sequence fragments randomly selected from the same species or from different species of the same genus, i.e. intra- or inter-species contaminations (Figure 1A). Unlike fragmentation and incompleteness, which caused uniform CG loss in all species, intra-species contamination led to different CG changes in different species (Supplementary Figure S6A). In about half species like *Burkholderia pseudomallei* and *K. pneumoniae*, a 4% contamination caused ~500 CG loss. However, there were no noticeable changes in *Helicobacter pylori* and *Streptococcus pyogenes*.

Intuitively, contamination will result in a larger pan-genome due to additional genes added, but should not reduce the CG number as no CGs are removed. The unexpected CG loss is further explained in the next section. However, as expected, in most species, the number of cloud genes (genes that are shared by <15% genomes, defined by Roary) increased constantly when more intra-species contamination was introduced (Supplementary Figure S6A). Furthermore, when inter-species contamination was introduced, the number of CGs dropped more slowly, whereas the number of cloud genes increased more rapidly (Supplementary Figure S6B).

Note only one original dataset was selected for the tests above. Choosing which 100 complete genomes to use as the original dataset to generate simulated MAGs may affect the pan-genome analysis results. To assess this effect, we created 50 original datasets for *E. coli*, each dataset with 100 randomly selected complete genomes. For each of the 50 original datasets, we further generated the fragmentation dataset (fragmented MAGs), the incompleteness dataset (fragmented + incomplete MAGs), and the contamination dataset (fragmented + incomplete + contaminated MAGs) (see Methods). The decrease of CG families in simulated MAGs was observed in every simulated dataset in *E. coli* (Figure 2E), *B. pertussis* (Figure 2F), and in other species (Supplementary Figure S7). Overall, fragmentation and incompleteness of MAGs lead to significant CG loss, while contamination has little effect on the core genomes but influences the accessory genomes.

### Core gene loss when using different tools and mixed datasets

In this section, to determine whether the CG loss was caused by the use of Roary, all the analyses were repeated by using BPGA [17] and Anvi’o [16] on 10 *E. coli* and 10 *B. pertussis* MAG datasets with the same gene models as used by Roary. Only Roary was used for mixed datasets (mixture of complete genomes and MAGs) (see Methods). A 90% SI and 100% CG definition thresholds were used in this section.

**Figure 3:**
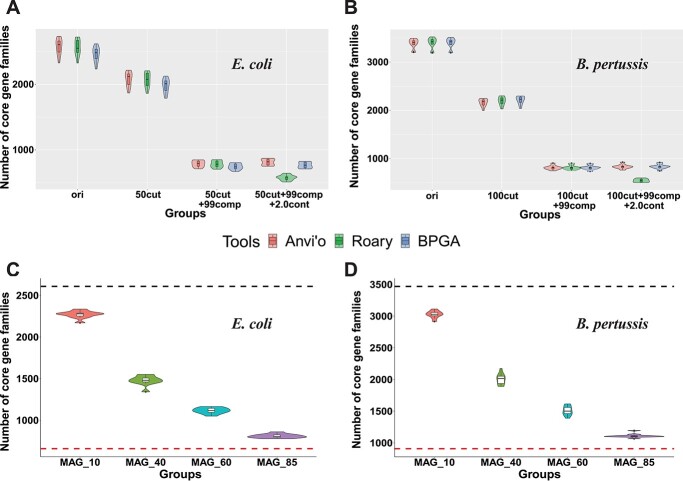
The core genome size change using different pan-genome tools and using mixed MAG datasets. (**A**) and (**B**) The violin plots of the number of CG families were calculated by Anvi’o (red), Roary (green) and BPGA (blue) in 10 *E. coli* datasets and 10 *Bordetella pertussis* datasets. See more details in legend in Figure 3E and F. (**C**) and (**D**) The number of CG families in *E. coli* and *B. pertussis* using mixed MAG dataset (see Methods). MAG_10 means that datasets contain 10% simulated MAGs and 90% complete genomes (see Figure 2B for examples). The black dot line indicates the number of CG families in the original dataset (100% complete genomes). The red dot line indicates the number of CG families in the simulation dataset (100% MAGs). All the core genomes were predicted by Roary, and each group contains 10 datasets.

Using different tools, the CG loss remained for fragmentation and incompleteness, but not for contamination (Figure 3A and B). In fact, in both *E. coli* and *B. pertussis*, with intra-species contamination the number of CGs increased slightly when using BPGA and Anvi’o, as we expected. We hypothesized that the opposite result by Roary (Figure 2E and F) was caused by possible bias in the gene clustering step of Roary. To test it, the CG sets before and after a 2% contamination were manually compared. The result indicated that some genes identified as CGs before contamination were misclassified as soft-CGs (genes present in >95% but <100% of the genomes) after contamination. Indeed, a manual comparison of the *E. coli* data (Supplementary Table S3 and Supplementary Figure S8) found that CGs in some genomes were clustered with non-CGs (e.g. from contamination sources) that share higher sequence identity to form a new gene family, leading to the loss of CG families. This should be an error unique in Roary. To summarize, CG loss remains when using different pan-genome analysis tools. Roary mistakenly reports CG loss when contaminations are in MAGs.

All the analyses so far used simulated data with 100% MAGs for pan-genome analyses. However, in reality a large proportion of published studies used mixed datasets in pan-genome analyses (Supplementary Figure S1E). To determine how much CG loss the mixed datasets may cause, four groups of mixed datasets containing from 10% to 85% MAGs were used in Roary analyses (see Methods). We found that the higher the MAG percentage is included, the more CGs will be lost (Figure 3C and D), which fits our expectation. To summarize, CG loss remains when using mixed MAG datasets; however, adding complete genomes into MAG datasets helps reduce CG loss.

### Core gene loss when using more relaxed core gene thresholds

The conclusion so far is that the CG loss will happen irrespective of what tools are used for MAG pan-genome analysis and what percentage of MAGs are included in a mixed dataset. The next question is that what should we do to avoid it. Two important parameters, CG definition threshold and SI, were tested to find a way to alleviate CG loss (see Methods for the definitions of CG and SI). In all the above analyses, the 100% CG threshold and 90% SI threshold were used. In this section, to determine the two parameters’ impacts on pan-genome results, different CG thresholds and SI thresholds were tested for simulated MAGs of *E. coli*.

**Figure 4:**
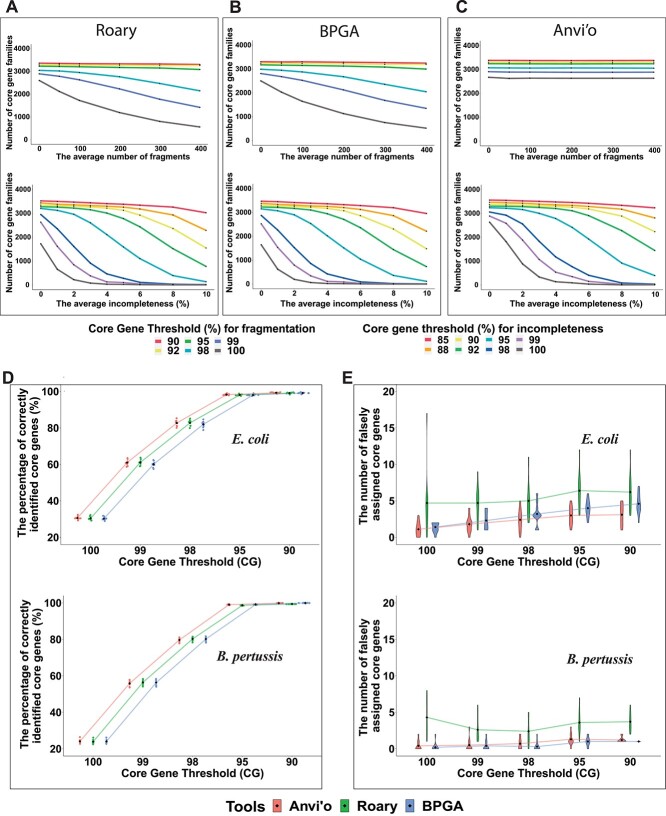
Lowering CG thresholds help alleviate the CG loss. (**A-C**) The numbers of CG families in simulated MAGs of *E. coli* (with different fragmentation and incompleteness levels) were predicted by using Roary, BPGA and Anvi’o using different CG thresholds. (**D**) The sinaplots show the impact of lowering CG thresholds in recovering true positives. (E) The violin plots show the impact of lowering CG thresholds in introducing false positives. Ten *E. coli* and *Bordetella pertussis* simulated MAG datasets with an average of 50/100 fragmentation and 1% incompleteness were used.

Firstly, the CG threshold was altered when the SI threshold was fixed (e.g. 90%). Under a specific fragmentation or incompleteness level, a lower CG threshold always gave a larger core genome size (Figure 4). This means the CG loss can be alleviated by lowering the CG threshold. However, the degree of the alleviation varied depending on what tools were used and how fragmented and incomplete the MAGs were. In Roary (Figure 4A) and BPGA (Figure 4B), a continuous CG loss was observed in more fragmented MAGs when using CG threshold $\ge$98%. In contrast, in Anvi’o with its default gene prediction method, more fragmented MAGs did not result in more CG loss, although choosing lower CG thresholds also resulted in higher CG numbers (Figure 4C). This is because by default Anvi’o uses Prodigal [56] in its metagenome mode for gene prediction, whereas BPGA and Roary use Prodigal (integrated in Prokka) in normal mode. Therefore, in Anvi’o, fragmentation had little effect on CG loss.

Compared to fragmentation, incompleteness caused more CG loss in all the three tools. This Even when the CG threshold is as low as 85%, for MAGs with an average incompleteness >10%, the CG loss was still a big problem for all the three tools (Figure 4A–C). Anvi’o was less affected (Figure 4C) but still suffers when incompleteness was $\ge$5% and CG threshold was $\ge$95%. Clearly a higher incompleteness will significantly reduce the size of the core genome regardless of what tools are used.

When using a lower CG threshold, some previous soft-CGs may now become CGs in both original and simulated datasets. Under the same CG threshold, a small number of CGs (<10) were found in only simulated MAGs but not in original complete genomes, and thus are false positives; CGs that were found in both complete genomes and simulated MAGs are true positives. With the CG threshold decreasing, the false positive rate rose slightly (Figure 4E), but the true positive rate rose very fast (Figure 4D), almost to 100% when the CG threshold $\le$95%.

In addition, in pan-genome analysis of complete genomes, the CG number will also increase with reduced CG thresholds. Hence, we have also studied how much difference there will be in terms of the increased CGs due to reduced CG thresholds between using original complete genome data and simulated MAG data. Supplementary Figure S9 depicts the calculation of the ‘coverage of increased CGs’ and ‘recovery rate of CGs.’ The result (Supplementary Table S4) shows that lowering the CG threshold caused much larger CG number increase in MAGs than complete genomes. This is expected because the simulated MAG data had a much smaller baseline (CG number = 782 with CG threshold = 100%), compared to the baseline of the complete genomes (CG number = 2669 with CG threshold = 100%). The large numbers of increased CGs in the simulated MAG data were due to the recovery of the lost CGs in the MAGs compared to complete genomes.

The ‘coverage of increased CGs’ relative to the complete genomes continuously went up as the CG threshold was reduced: it was 97.19% when the CG threshold was set at 90% (Supplementary Table S4). More importantly, the ‘recovery rate of CGs’ (Supplementary Figure S9) relative to the complete genomes baseline is the recall, and that relative to the MAGs is the precision. Supplementary Table S4 shows the change of the recall and precision with reduced CG threshold. The *F*-score was the best (90.1%) when the CG = 95%.

To summarize, using a lower CG threshold based on the quality of MAGs helped recover the lost CGs with a very low false positive rate and high CG recovery rate. In contrast, changing the SI threshold (with fixed CG threshold) had very little help in recovering lost CGs in simulated MAGs (Supplementary Figure S10).

### Functional and phylogenetic analysis

In pan-genome analysis, after the core and accessory genes are identified, downstream analyses are often performed on these genes to understand the genome phylogeny and gene functions. In addition, functional enrichment analysis is often performed on core, accessory, and unique genes to explain species adaptation to various environments. Given that the core genome size is inevitably decreased in the pan-genome analysis of MAGs, there will be some consequences in the COG functional predictions for the CGs and genome phylogeny construction. In this section, we used 10 *E. coli* and 10 *B. pertussis* MAG datasets to study the effects on functional and phylogenetic analysis.

**Figure 5:**
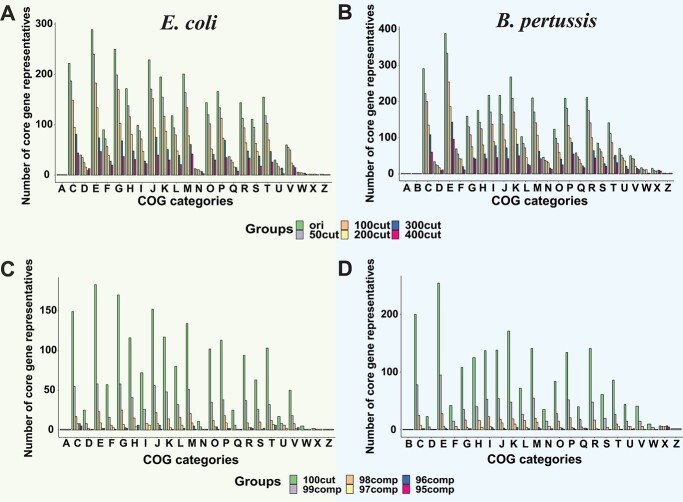
The COG analysis of CGs in simulated MAGs. (**A**) and (**B**) The bar plots of the number of CGs with different fragmentation levels in each COG category. (**C**) and (**D**) The bar plots of the number of CGs with different incompleteness levels in each COG category. COG category single letter codes: A: RNA processing and modification, C: energy production and conversion, D: cell cycle control and mitosis, E: amino Acid metabolism and transport, F: nucleotide metabolism and transport, G: carbohydrate metabolism and transport, H: coenzyme metabolism, I: lipid metabolism, J: translation, K: transcription, L: replication and repair, M: cell wall/membrane/envelop biogenesis, N: cell motility, O: post-translational modification, protein turnover, chaperone functions, P: inorganic ion transport and metabolism, Q: secondary structure, R: general functional prediction only, S: function unknown, T: signal transduction; U: intracellular, trafficking and secretion, V: defense mechanisms, W: extracellular structures, X: mobilome: prophages, transposons, Z: cytoskeleton.

When using the 100% CG threshold, the number of CGs assigned to COG categories was dramatically decreased with increasing fragmentation and incompleteness (Figure 5). Incompleteness (Figure 5C and D) had a more significant impact than fragmentation (Figure 5A and B). When using the 100% CG threshold, the COG functional enrichment in the core and accessory genes were also significantly changed due to fragmentation and incompleteness (Supplementary Figure S11A–C), leading to inaccurate inferences to explain environmental adaptation. As expected, when using lower CG thresholds, CG functional predictions were slightly affected by fragmentation and incompleteness (Supplementary Figure S12). Moreover, the COG functional enrichment analysis of core and accessory genes were also less affected (Supplementary Figure S11D–F). However, the accuracy loss could not be fully eliminated especially for MAGs with higher incompleteness. It is also likely that the inclusion of falsely predicted CGs may lead to incorrect functional analysis results.

**Figure 6:**
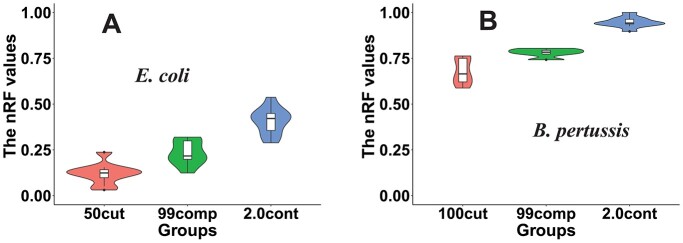
Simulated MAGs cause significant changes in the phylogenetic trees constructed based on core genome alignment. (**A**) and (**B**) The violin plots of the nRF distance values (calculated by ETE3 toolkits) between MAG trees and complete genome trees.

Pan-genome analysis tools produce a CG set, and phylogenetic trees are often built based on the single-copy CG alignment to delineate the evolutionary relationship among the studied genomes. To quantify the topological changes between the trees of original genomes and simulated MAGs, three metrics were calculated: (i) the normalized Robinson-Foulds (nRF) distance [53], (ii) the generalized RF (gRF) distance [54] and (iii) the percentage of shared branches [53]. The higher the nRF and gRF values, the more differences between the trees. For example, the nRF values increased from 0.1 in simulated MAGs with only fragmentation to 0.4 in simulated MAGs with fragmentation + incompleteness + contamination in *E. coli* datasets (Figure 6A). The trend was the same in *B. pertussis* datasets except that nRF values increased from 0.6 to 0.9 (Figure 6B). This was also supported by the percentage of shared branches (Supplementary Figure S13A and B) and the gRF distance (Supplementary Figure S13C and D).

In addition to the single-copy CG tree, pan-genome analysis tools often also produce a phylogeny based on a gene presence and absence matrix. Comparing to the complete *E. coli* genomes (Supplementary Figure S13E), a dramatic shrinkage in the CG area was observed in the gene presence and absence dot plot of simulated *E. coli* MAGs (Supplementary Figure S13F). All these findings indicate that the more fragmentation, incompleteness and contamination MAGs have, the less accurate the phylogeny will be, irrespective of using CG alignment or gene presence and absence for tree construction.

## Discussion

Concerns have been raised that mis-assembly and mis-binning can be very common in the automatically generated MAGs [25, 36, 37, 57, 58]. In this study, we aimed to critically evaluate the accuracy loss of using MAGs in pan-genomics. We approached this goal by comparing the pan-genome analysis results of complete genomes and simulated MAGs. Our simulation considers fragmentation, incompleteness and contamination (Figure 1), and follows the empirical distribution (Supplementary Figure S2) of real MAGs from human gut microbiome [38].

Our major result is that the CG loss caused by fragmentation and incompleteness in MAGs is universal, regardless of what species is used (Figure 2 and Supplementary Figure S4), what pan-genome analysis software is used (Figure 3), what CG threshold is used (Figure 4), and what fraction of MAGs is included (Figure 3). However, to what extent the CG can be lost in MAGs varies among species, softwares, CG thresholds and fractions of MAGs. The most important finding is that lowering the CG threshold and choosing the metagenome mode for gene prediction (as Anvi’o does) can alleviate the CG loss (Figure 4A–C, Supplementary Table S4) with minimal false positives (Figure 4D and E).

**Figure 7:**
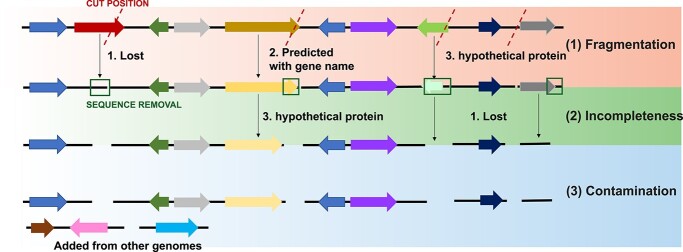
Incorrect gene predictions caused by lost genes or falsely predicted genes due to fragmentation, incompleteness and contamination. Arrows shown in different colors represent predicted genes in genome sequences.

Why fragmentation, incompleteness and even contamination can cause CG loss? Previous studies have shown that pan-genome analysis suffers from incomplete, inconsistent and inaccurate gene predictions in bacterial isolate genomes [21, 59]. Therefore, the CG loss in MAGs must be directly caused by missing genes in the gene prediction step. Shown in Figure 7, we delineated the process of CG loss with fragmentation, incompleteness and contamination. The result of these is that gene prediction programs will fail to call the affected genes, or call an incorrect gene due to reading frame shifts, leading to falsely predicted genes [60]. Contamination from closely related strains or species should not lead to CG loss due to no removal of existing genomic regions. However, the size of the core genome still decreased when using Roary, which was not seen in BPGA and Anvi’o (Figure 3A and B). It appears that some CG clusters (families) before contamination were falsely split into multiple gene clusters in Roary, leading to the loss of CGs. Gene clusters being incorrectly split into multiple smaller clusters were also noticed in other studies [21, 59], which suggested that the removal of contamination and annotation errors are essential to construct an accurate pan-genome [59].

Two possible limitations exist in this work. The first one is the lack of a rigorous pre-screening step of the original genomes used for simulation studies. Recent papers [61, 62] have suggested that it is important to remove the confounding strains (e.g. mis-classified, genome-reduced, chimeric, and multiple clonal and highly similar strains) for a more accurate pan-genome analysis. Because we only used completely assembled RefSeq isolate genomes (no gaps, no contaminations, no chimeric genomes), this pre-screening step is likely not necessary and will not affect our conclusions. All our findings are supported by analyses on 17 species (Supplementary Figure S4) and each species with multiple randomly selected 100-genome datasets (Figure 2E and F).

The second limitation is that the simulation method shown in Figure 1A may not fully reflect the generation process of real MAGs from read assembly and binning. For example, fragmentation and incompleteness are not separatable because they most likely happen together in read assembly. However, this will not change our main findings at all. More importantly, mis-assembly and mis-binning in real MAGs are not completely random, and not all genes are equally impacted by fragmentation and incompleteness. For example, the CGs should be less affected than accessory genes (e.g. prophages, insertion sequences and other mobile genetic elements) in mis-assembly and mis-binning. Therefore, the CG loss in this study may be overestimated due to random completeness loss and random contamination addition in our simulated MAGs.

To address this concern, we have found a real MAG data and its corresponding isolate genomes from Meziti *et al.* [36]. These real data contain five pathogenic *E. coli* isolate-MAG pairs from human fecal samples: E158-MG24, E124-MG23, B45-MG6, E184-MG19 and B200-MG15. These real MAGs (e.g. MG24) were generated in [36] by using metagenomic read assembly and binning from the same samples as the isolate genomes (e.g. E158). Each pair of the five isolate-MAG pairs share >99.8% ANI (average nucleotide identity) supporting that the isolate genome and the MAG are indeed a pair. We have mixed the five *E. coli* real MAGs with a varying number of *E. coli* complete genomes, and similarly mixed their corresponding isolate genomes with *E. coli* complete genomes. Thus, we have built four mixed-MAG datasets with different percentages of MAGs and four corresponding mixed-isolate genome datasets. Comparing the core genomes of mixed-MAG dataset and mixed-isolate genome dataset (Supplementary Figure S14) confirmed the main findings that were made in our simulation studies: (i) there is a significant CG loss (~25% if using CG threshold = 100%) in real MAGs compared to their corresponding isolate genomes, irrespective which pan-genome analysis tools are used; (ii) when mixing a higher percentage of complete genomes, the CG loss alleviated; and (iii) when using a more relaxed CG threshold = 90%, the CG loss alleviated. Overall, the CG loss remains to be true in the real MAG data.

Finally, we provide recommendations to deal with CG loss in pan-genomics of MAGs based on our findings and evaluation:

(1). The loose CG threshold (i.e. genes present in 95% or 90% genomes) instead of the strictest one (100%) should be used for MAGs. According to Figure 4, a higher CG threshold like 95% can be applied to MAGs with only low fragmentation and low incompleteness. A lower CG threshold like 90% or 85% should be used for MAGs with higher fragmentation (average number of fragments >100), and higher incompleteness (average incompleteness >5%). Although the clustering sequence identity is also an important parameter in pan-genome analysis, changing the threshold of it did not affect the CG counts in MAGs (Supplementary Figure S10).

(2). It is important to use gene prediction programs that can handle fragmented genes, for instance, using the metagenome mode of Prodigal. Some pan-genome analysis tools call gene prediction programs internally (e.g. Anvi’o), while others demand users to provide externally predicted genes as input (e.g. Roary and BPGA). This study primarily used Prokka to prepare predicted genes as input to Anvi’o, Roary and BPGA. However, when Anvi’o was run with its default gene prediction method (metagenome mode of Prodigal), the CG loss due to increasing fragmentation can be almost negligible (Figure 4).

(3). Use mixed MAG datasets including complete or near complete isolate genomes (if available) rather than only MAGs for pan-genome analysis. The CG loss can be practically alleviated by combining complete genomes with MAGs.

(4). Some very recent pan-genome analysis tools, e.g. Panaroo [59] and GenAPI [63] have been developed to tackle the problems of mis-assembly and mis-annotation in low-quality draft isolate genomes. They could be very useful alternatives to those older but more popular tools (Supplementary Figure S1) for analyzing MAGs, even although they were not designed for MAGs. Future research should include these newer tools in the evaluation of pan-genome analysis of MAGs.

**Key Points**

- Fragmentation and incompleteness of MAGs lead to significant core gene (CG) loss, while contamination influences the accessory genomes.
- In the pan-genome analysis using MAGs, a lower CG threshold (90% or 95%) should be used to alleviate core genome loss, and thus improve gene functional enrichment analysis.
- Anvi’o works better on fragmented MAGs, whereas Roary performs worst on MAGs with contamination.
- Combining complete isolate genomes with MAGs for pan-genome analysis is better than using only MAGs.

## Supplementary Material

Supplementary_Figures_bbac413

Table_S1_bbac413

Table_S2_bbac413

Table_S3_bbac413

Table_S4_bbac413

Supplementary_Methods_bbac413

Supplementary_Results_bbac413

## Data Availability

All the computer codes and data generated in this study have been made available at GitHub (https://github.com/tli14/PanMAGs).

## Author Biographies

**Tang Li** is a graduate student of UNL. She works on bioinformatics analysis of bacterial pan-genome and metagenome.

**Yanbin Yin** is an Associate Professor of UNL. He works on bioinformatics tool development to understand genome biology and evolution.
