# Supplementary material for: Critical assessment of pan-genomic analysis of metagenome-assembled genomes
Source: Brief Bioinform. 2022 Sep 17;23(6):bbac413. doi: 10.1093/bib/bbac413 (PMC9677465; doi:10.1093/bib/bbac413)
Supplement: Supplementary_Figures_bbac413 [file supplementary_figures_bbac413.docx]

**Supplementary Figures**

Figure S1. Summary of 39 publications that used MAGs in pan-genome analysis. (**A**): The bar plot shows the publication years. (**B**): The pie chart shows the ecological environments of analyzed MAGs. (**C**): The bar plot shows the pan-genome analysis tools (multiple tools used in a same study were counted separately). (**D**): The pie chart shows the CG thresholds used in pan-genome studies. (**E**): The bar plot to survey the percentages of MAGs used in pan-genome analysis (y-axis is the count of pan-genomes, and x-axis is the percentages of MAGs). (**F**): The bar plot to survey the numbers of genomes/MAGs used in pan-genome analysis (y-axis is the count of pan-genomes, and x-axis is the numbers of genomes/MAGs).

**Figure S2. The Pan-genome composition of 17 species constructed by using Roary.** The n beside each bar represents the number of complete genomes used for building species pan-genome. Roary was run with parameters (-i 90 -cd 100 -s -e -n), which produced four classes of genes shown in different colors. Some terms (e.g., cloud genes and shell genes) are not often seen from other pan-genome analysis tools.

**Figure S3. The statistical analysis of UHGG MAGs.** (**A**) The distribution histogram of contig numbers, completeness percentage, and contamination rates in 276,349 MAGs. (**B**) The density plots for UHGG MAGs of five species: EC for *Escherichia coli* (n=4,391), EF for *Enterococcus faecium* (n=333), KP for *Klebsiella pneumoniae* (n=641), PA for *Pseudomonas aeruginosa* (n=63), and SA for *Staphylococcus aureus* (n=57).

**Figure S4. Fragmentation and incompleteness effect on the number of core gene families**. (**A**) The core genome size continuously decreases as the simulated MAGs become more fragmented. The red curve was predicted using an exponential model for the correlation between the x-axis (the number of fragments) and the y-axis (the core genome size). (**B**) The core genome size decreases more rapidly as the simulated MAGs become less complete.

**Figure S5. Fragmentation simulation based on average fragment length**. The violin plot of the core genome sizes in 10 *E. coli* or *B. pertussis* original datasets and their corresponding simulated MAG datasets. Groups: “ori” represents the original datasets; “100kbp”, “50kbp”, “20kbp” and “10kbp” represent that the average length of fragments in the simulated MAGS is 100, 50, 20 and 10 kilobase pair (kbp).

**Figure S6. Contamination effects on the number of core and cloud gene families.** (**A**) The number of core gene families (colored curves, left y-axis) and cloud gene families (black curves, right y-axis) in 100 MAGs with a different average intraspecies contamination rate (%). (**B**) The interspecies and intraspecies contamination comparison. Group labeled as genus represents the interspecies contamination from other species in the same genus, and group labeled as species represents the intraspecies contamination from different strains in the same species.

**Figure S7.** Core genome sizes decrease in *S. aureus* and *K. pneumoniae*. (A) The violin plot of the core genome sizes in 30 *S. aureus* original datasets and their corresponding simulated MAG datasets. (B) The violin plot of the core genome sizes in 30 *K. pneumoniae* original datasets and their corresponding simulated MAG datasets. See more details in legend in **Figure 2E and F**.


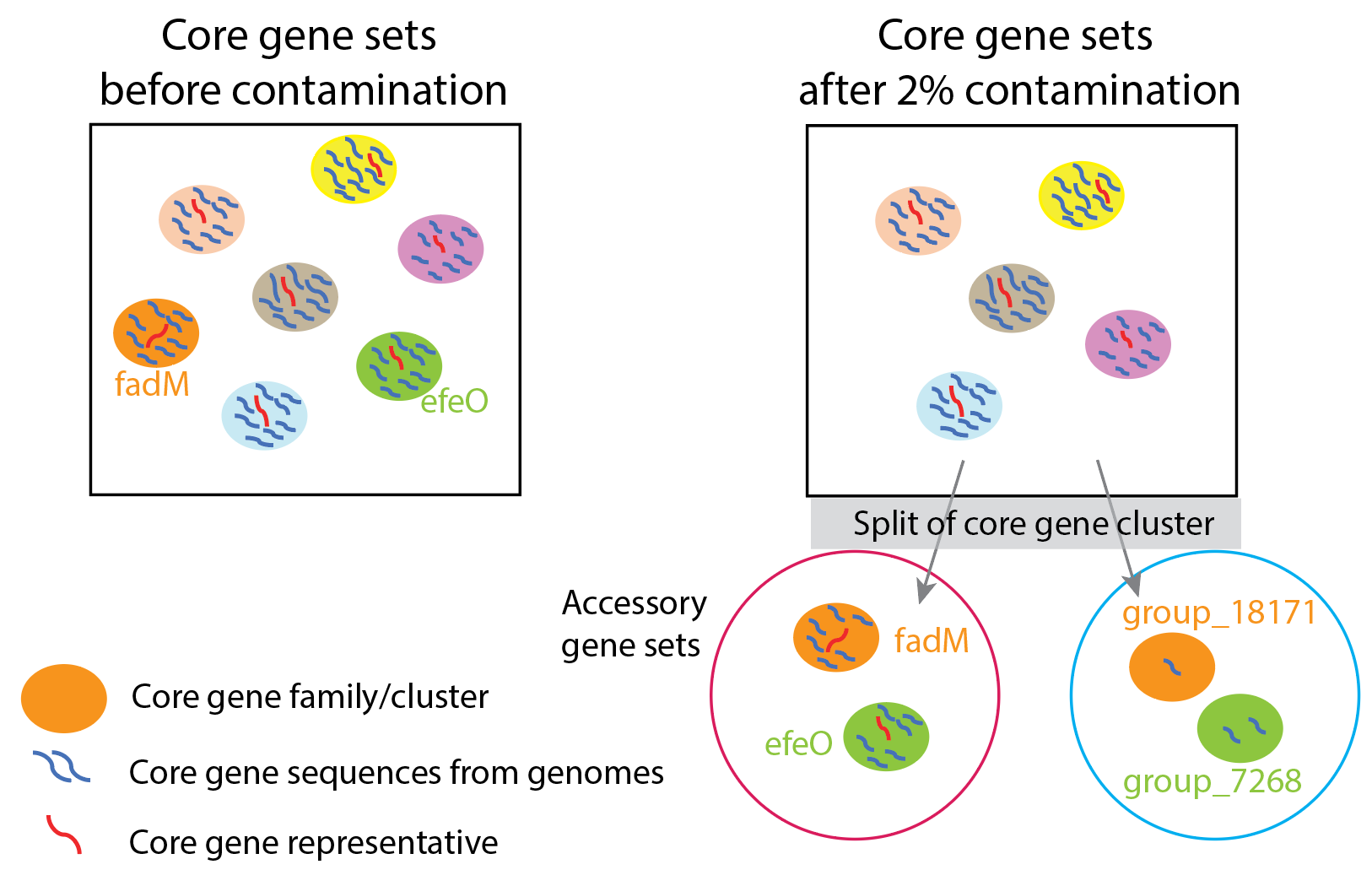


**Figure S8. A schematic illustration of core gene loss due to an error in Roary gene clustering step.** fadM and efeO are two example genes whose gene clusters were split into two smaller gene clusters after contamination was added. This error leads to the loss of the two core gene clusters, which is not found in BPGA and Anvi’o.


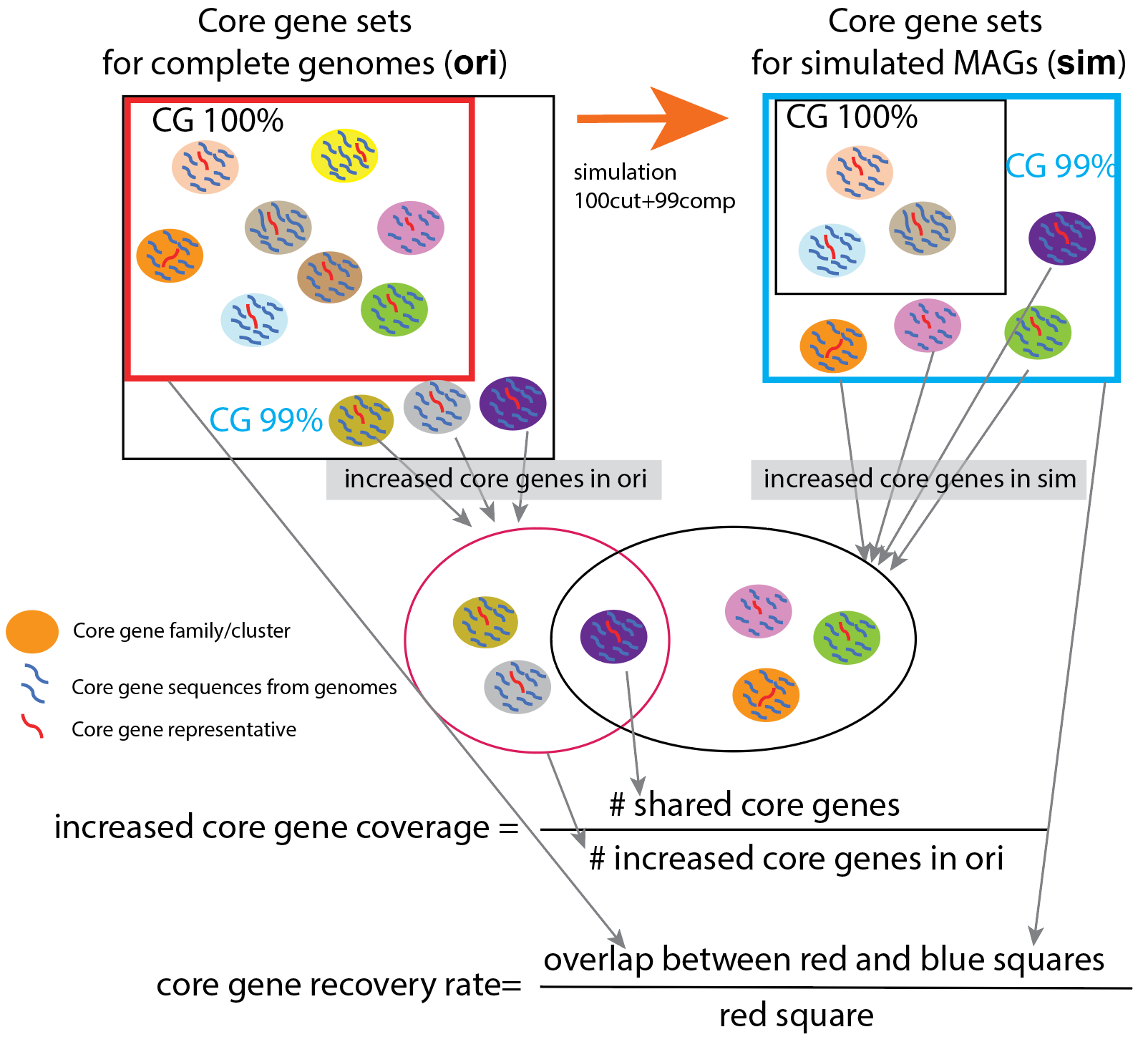


**Figure S9. A schematic illustration of increased core gene coverage and core gene recovery rate calculation**. Compared to the core gene set under the 100% core gene threshold (CG 100%), some new gene clusters were included in the core gene set when using the lower core gene threshold (e.g., CG 99%). These “increased core genes” will occur in both the original dataset (complete genomes) and simulated MAG dataset when using a lower CG threshold. If these increased core gene clusters are found in both datasets (ori and sim), they are "shared core genes". The increased core gene coverage was calculated by using the number of shared increased core genes divided by the increased core genes in ori dataset. The core gene recovery rate (sensitivity) was calculated by using the shared core genes divided by the total core genes in ori dataset at 100% CG.

**Figure S10. Different gene sequence identity comparison.** Line plots of the number of core gene families in *E. coli* MAG datasets with different incompleteness percentages. The core gene (CG) threshold used is 100% (**A**), 99% (**B**), 95% (**C**) and 90% (**D**). Different colors represent the minimum identity percentage for gene clustering. In (A) and (B), the four curves almost have no difference and overlap very much.

**Figure S11.** **Enrichment of COG functional categories in *E. coli* core, accessory and unique genes compared to the pan-genome.** Bar plots show the enrichment of COG categories in each gene group as P-values (-log10) calculated with binomial test (P-value<0.05). The core gene threshold 100% (**A-C**) and 95% (**D-F**) are used in original datasets and their corresponding simulated MAG datasets. See more details in legend in **Figure 3** and **Figure 6.**

**Figure S12. Core gene thresholds influence COG analysis for *E. coli* core genome.** Bar plots of the number of core gene representatives in *E. coli* datasets with different fragmentation (left) and incompleteness (right) in each COG category. The core gene threshold 99% (**A**), 98% (**B**) and 95% (**C**) are used. See more details in legend in **Figure 6.**


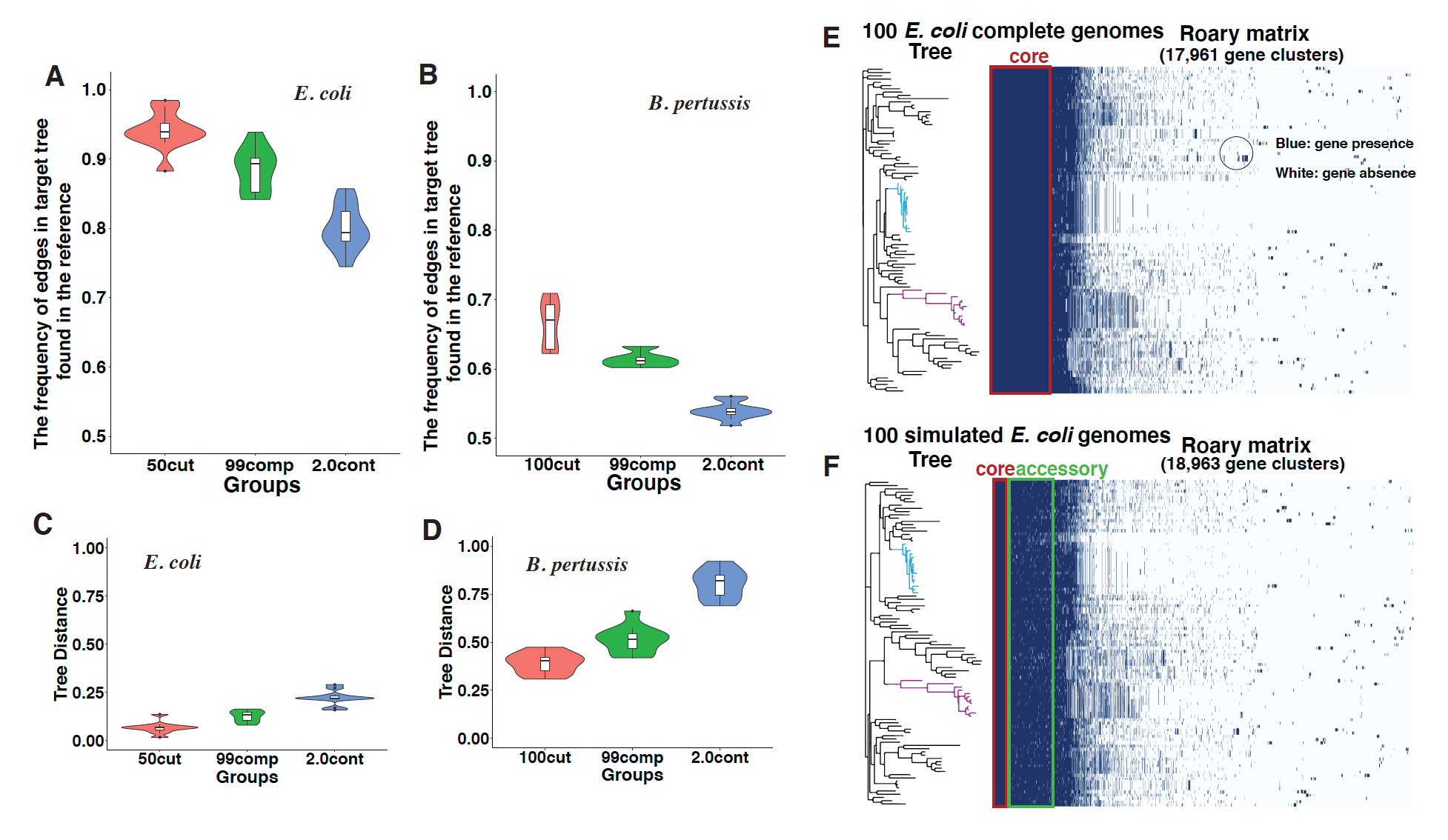


**Figure S13. The effects on phylogenetic trees in different species.** (**A**) and (**B**) The violin plot of the percentage of tree branches shared between MAG trees and the complete genome tree constructed based on core genome alignment. For each group, 10 *E. coli* or *B. pertussis* datasets were used. (C) and (D) The violin plots of the gRF distances (calculated by TreeDist) between MAG tree and complete genome tree. (E) The phylogenetic tree and Roary gene presence and absence matrix (as a dot plot) for 100 *E. coli* complete genomes. (F) The phylogenetic tree and Roary gene presence and absence matrix (as a dot plot) for 100 *E. coli* simulated MAGs with an average of 100 fragments and 1% incompleteness. The phylogenetic tree in (E) and (F) were created using the binary presence and absence of accessory genes. In the dot plot, each row corresponds to a branch (one genome or MAG) in the tree, and each column represents a gene family/cluster. White indicates gene absence and blue indicates gene absence.


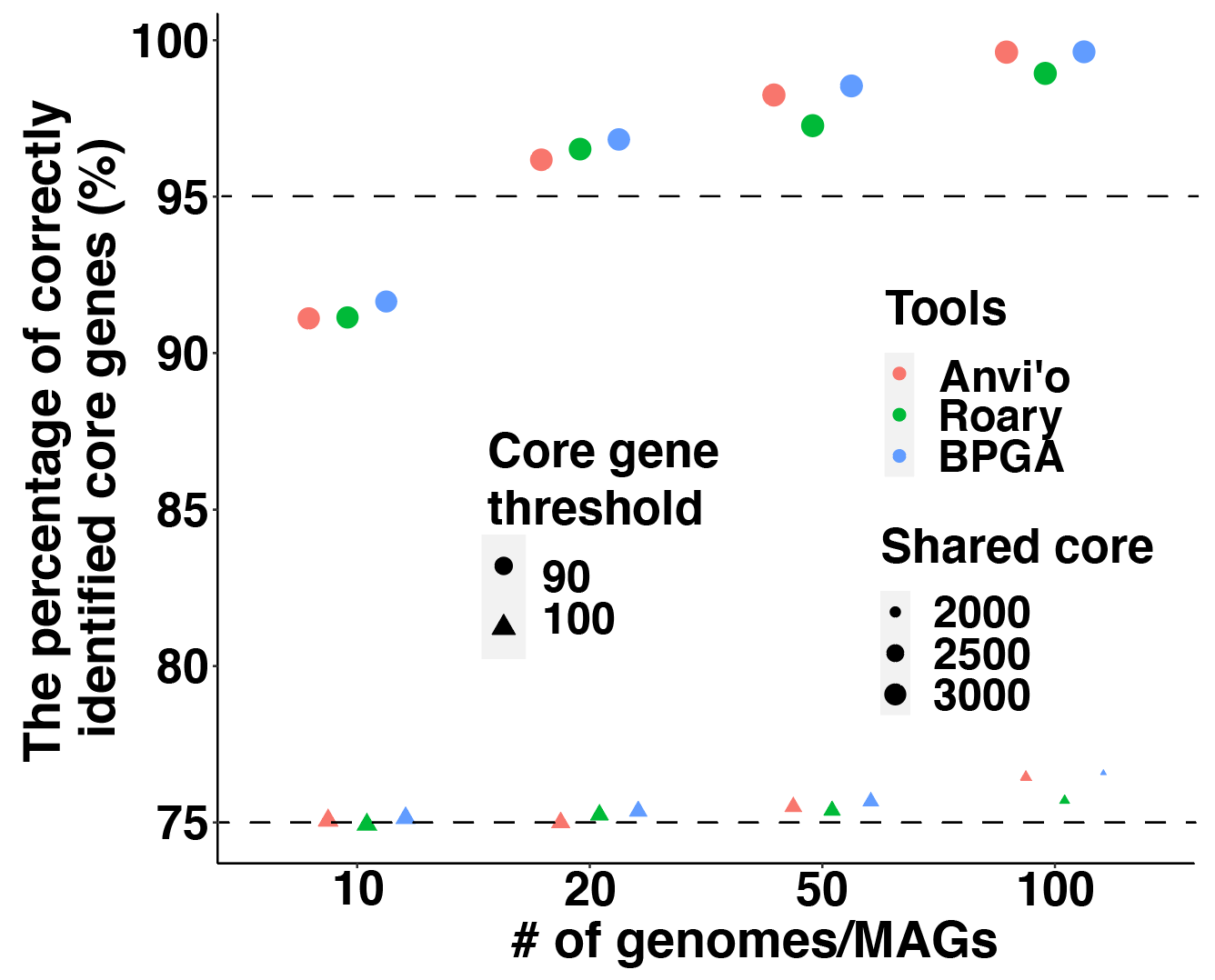


**Figure S14. Comparison of core genes in real MAG datasets and their corresponding isolate genome datasets.** The x-axis is the number of genomes in the datasets. Each dataset contains 5 real *E. coli* MAGs or 5 *E. coli* isolate genomes (of the same isolate-MAG pair), and the rest are complete genomes. For example, “10” means a dataset of 5 real MAGs (or isolates) plus 5 complete genomes; “100” means a dataset of 5 real MAGs (or isolates) plus 95 complete genomes. The y-axis shows the percentages of correctly identified core genes between MAG dataset and isolate dataset, calculated by the number of shared core genes in MAG dataset and isolate dataset (isolate - MAG) divided by the total number of core genes in isolate dataset.
