# Supplementary material for: Critical assessment of pan-genomic analysis of metagenome-assembled genomes
Source: Brief Bioinform. 2022 Sep 17;23(6):bbac413. doi: 10.1093/bib/bbac413 (PMC9677465; doi:10.1093/bib/bbac413)
Supplement: Supplementary_Methods_bbac413 [file supplementary_methods_bbac413.docx]

**Supplementary Methods for**

**Critical assessment of pan-genomic analysis of metagenome-assembled genomes**

*Tang Li^1^, Yanbin Yin^1,$^*

*^1^Nebraska Food for Health Center, Department of Food Science and Technology, University of Nebraska - Lincoln, Lincoln, NE, 68508, USA*

^$^corresponding author

Yanbin Yin

Tel: 1-402-472-4303

Email: [yyin@unl.edu](mailto:yyin@unl.edu)

## **Literature search of pan-genome analysis with MAGs**

We have searched in PubMed and Google Scholar using the keyword query: ("pangenome" OR "pan-genome") AND ("metagenome-assembled genome" OR "MAGs") in titles and abstracts. Papers were kept if the following criteria were met: (i) studied organism(s) were prokaryotes, (ii) at least one MAG was used in pan-genome analysis, and (iii) a specific pan-genome analysis tool and specific parameters were described. Based on these results, we selected the pan-genome tools to be tested, the parameters to be used in pan-genome analysis and the dataset size to create.

**Distributions of fragmentation, completeness, and contamination of real MAGs**

To perform more realistic MAG simulation, a total of 276,349 MAGs of the Unified Human Gastrointestinal Genome (UHGG) collection [1] were used to determine the distribution of contig number, completeness, and contamination in MAGs. Specifically, the MAG summary file in the MGnify FTP site ([ftp.ebi.ac.uk/pub/databases/metagenomics/mgnify_genomes/human-gut/v1.0/genomes-all_metadata.tsv](ftp://ftp.ebi.ac.uk/pub/databases/metagenomics/mgnify_genomes/human-gut/v1.0/genomes-all_metadata.tsv)) was processed. Histograms and density plots were created using R ggplot2 [2].

**Simulate MAGs from complete genomes**

In this section, we introduced how MAGs were simulated from the complete genomes resembling the distribution of fragmentation, completeness, and contamination observed in UHGG MAGs.

For example, to simulate a dataset of 100 MAGs with an average fragment number of 50, a Python script was developed to generate 100 random numbers with a mean = 50 and following an F-distribution. The F-distribution was selected because it fitted the distributions observed in real UHGG MAGs. These 100 random numbers corresponded to the numbers of cuts that were made in the 100 complete genomes to create a *simulated MAG dataset* (100 fragmented genomes). Simulated MAG dataset with incomplete genomes and contaminated genomes were generated in the same manner. The three operations were combined for MAG simulations and depicted in **Figure 1A**.

We have also used the average fragment length for fragmentation simulation to compare with the simulation using average fragment number. There were four groups of average fragment length: 10, 20, 50, and 100 kilobase pair (kbp). For example, to simulate a dataset of 100 MAGs with an average fragment length of 10 kbp, a Python script was used for each complete genome to generate a list of random numbers with the average = 10,000 and following the normal distributions, representing the length of fragments in the genome.

Fragments from genomes of the same species or genus were added as contamination. This is because contamination in real MAGs is often introduced at the contig binning step, which is based on the fact that DNA fragments of closely related genomes (e.g., of the same species/genus) tend to share more similar nucleotide compositions. The species-level contamination was added from complete genomes within the same species, whereas the genus-level contamination was chosen from genomes of other species within the same genus.

**Using real datasets containing *E. coli* MAGs and isolate genomes**

In this section, we explained how to use MAG data and its corresponding isolate genomes to generate realistic datasets to test results oberseved in simulated MAG datasets.

Firstly, five of eight pathogenic *E. coli* isolates from diarrheal samples and their corresponding MAGs were downloaded from NCBI. The five isolate-MAG pairs used were: E158-MG24, E124-MG23, B45-MG6, E184-MG19, and B200-MG15. Three isolate-MAG pairs were removed due low pairwise ANI and data unavailability. MAGs were downloaded under BioProject number PRJNA486009 released by Meziti A. *et al.*[3], while isolate genome assemblies were found by their strain name. The pairwise ANI for each isolate-MAG pair was evaluated by FastANI [4].

To test the effects caused by MAGs in datasets with different sizes, four datasets containing varying numbers of genomes (10, 20, 50 and 100) were used. For example, the 10-genome MAG dataset contains the 5 *E. coli* MAGs downloaded above and 5 *E. coli* complete genomes randomly selected from the original dataset. The 10-genome isolate dataset contains the 5 *E. coli* isolates and the same 5 complete genomes used in the MAG dataset. All MAGs, isolates and complete genomes were annotated by Prokka [5]. Pan-genome analyses were performed for each dataset by using three tools with the same parameters as mentioned above. The core gene set shared by MAG and isolate datasets was compared among three tools when using CG 100% and CG 90%.

References:

1. Almeida A, Nayfach S, Boland M, et al. A unified catalog of 204,938 reference genomes from the human gut microbiome. Nat. Biotechnol. 2021; 39:105–114

2. Wickham H. ggplot2: Elegant Graphics for Data Analysis. Springer-Verlag New York 2016;

3. Meziti A, Rodriguez-R LM, Hatt JK, et al. The Reliability of Metagenome-Assembled Genomes (MAGs) in Representing Natural Populations: Insights from Comparing MAGs against Isolate Genomes Derived from the Same Fecal Sample. Appl. Environ. Microbiol. 2021; 87:1–15

4. Jain C, Rodriguez-R LM, Phillippy AM, et al. High throughput ANI analysis of 90K prokaryotic genomes reveals clear species boundaries. Nat. Commun. 2018; 9:5114

5. Seemann T. Prokka: Rapid prokaryotic genome annotation. Bioinformatics 2014; 30:2068–2069
