# Supplementary material for: Critical assessment of pan-genomic analysis of metagenome-assembled genomes
Source: Brief Bioinform. 2022 Sep 17;23(6):bbac413. doi: 10.1093/bib/bbac413 (PMC9677465; doi:10.1093/bib/bbac413)
Supplement: Supplementary_Results_bbac413 [file supplementary_results_bbac413.docx]

**Supplementary Results for**

**Critical assessment of pan-genomic analysis of metagenome-assembled genomes**

*Tang Li^1^, Yanbin Yin^1,$^*

*^1^Nebraska Food for Health Center, Department of Food Science and Technology, University of Nebraska - Lincoln, Lincoln, NE, 68508, USA*

^$^corresponding author

Yanbin Yin

Tel: 1-402-472-4303

Email: [yyin@unl.edu](mailto:yyin@unl.edu)

**MAGs are increasingly used in pan-genome analyses**

Here, we summarized the literature search results to show the trend of using MAGs for pan-genome analysis. There was an increasing use of MAGs in pan-genome analyses since 2017 (**Figure S1A**). A total of 39 publications were found, however, this number is certainly underestimated as our literature search applied very strict conditions (see **Supplementary Methods**). These analyzed MAGs were from various ecological environments (**Figure S1B**). The most popular pan-genome analysis tools were Anvi’o [1] and Roary [2] (**Figure S1C**), while other tools such as BPGA [3], GET_HOMOLOGUES [4], and OrthoMCL [5] were also used. The strictest core gene (CG) threshold 100% (see **Methods**) was used in 2/3 studies (**Figure S1D**). In some studies, only MAGs were used in the pan-genome analyses (MAG% = 100% in **Figure S1E**), e.g., the studies of hydrothermal vents [6,7], ocean [8], and animal gut [9]. However, in others a mixture of complete isolate genomes and MAGs were used (MAG% < 100% **Figure S1E**), e.g., in human gut [10,11]. Some studies analyzed pan-genomes of thousands of genomes/MAGs (**Figure S1F**), while most studies analyzed < 100 genomes/MAGs. The details about these publications are provided in **Table S1**.

**Simulated MAGs from complete genomes**

To guide the creation of simulated MAGs from complete genomes, we have plotted the distributions of contig number, genome completeness, and contamination in 276,349 UHGG (Unified Human Gastrointestinal Genome) MAGs (**Figure S2A)** [38]**.**

The number of contigs in these real MAGs varied from 1 to 2282 with a mean = 208. The completeness ranged between 50% and 100% with a mean = 85.18%, while the contamination rate varied from 0% to 5% with a mean = 1.2%. These three metrics all have skewed distributions, even when they were plotted for individual species (**Figure S2B**). Therefore, the F-distribution, a theoretical distribution in Statistics used to model skewed distributions, was applied to guide the creation of simulated MAGs from complete genomes (see **Methods** and **Figure 1A**).

**Pan-genome analysis of complete bacterial genomes**

Before the MAG simulation, the pan-genome of each species were constructed by using all complete genomes of the species to select representative species. For 17 species used in this study, they belong to 15 taxonomic families of three phyla: Proteobacteria (10 species), Firmicutes (5) and Actinobacteria (2) (**Table S2)**. The pan-genome size (**Table S1**, **Figure S2**) was positively correlated with the average genome size (Pearson correlation coefficient R = 0.72, p = 0.0012) [12,13] and the number of genomes used (R = 0.71, p = 0.0013). One well-known fact about pan-genome analysis is that some bacterial species has more open pan-genome than others [14,15]. The fitting parameter exponent γ in Heaps’ law model was often used to predict the openness and closeness of the pan-genome [14,16]. When γ >0, the pan-genome is open, and its size increases as more genomes are used. The γ values shown in **Table S2** are consistent with previous findings [17–19]: the two largest γ values were found in *Salmonella enterica* and *Escherichia coli*, while three species (*Bordetella pertussis*, *Mycobacterium tuberculosis*, and *Corynebacterium pseudotuberculosis*) only had γ values at ~0.03. The lower γ values indicate that their pan-genomes were near to be closed. Since *E. coli* and *B. pertussis* not only have larger numbers of complete genomes but also have extreme differences in ANI values, pan-genome structures (the relative proportion of core, accessory, and unique genes), and γ values, they were selected as representative species in this study.

1. Eren AM, Esen OC, Quince C, et al. Anvi’o: An advanced analysis and visualization platformfor ’omics data. PeerJ 2015; 3:e1319

2. Page AJ, Cummins CA, Hunt M, et al. Roary: Rapid large-scale prokaryote pan genome analysis. Bioinformatics 2015; 31:3691–3693

3. Chaudhari NM, Gupta VK, Dutta C. BPGA-an ultra-fast pan-genome analysis pipeline. Sci. Rep. 2016; 6:24373

4. Contreras-Moreira B, Vinuesa P. GET_HOMOLOGUES, a versatile software package for scalable and robust microbial pangenome analysis. Appl. Environ. Microbiol. 2013; 79:7696–7701

5. Li L, Stoeckert CJ, Roos DS. OrthoMCL: Identification of ortholog groups for eukaryotic genomes. Genome Res. 2003; 13:2178–2189

6. Anderson RE, Reveillaud J, Reddington E, et al. Genomic variation in microbial populations inhabiting the marine subseafloor at deep-sea hydrothermal vents. Nat. Commun. 2017; 8:1114

7. Moulana A, Anderson RE, Fortunato CS, et al. Selection Is a Significant Driver of Gene Gain and Loss in the Pangenome of the Bacterial Genus Sulfurovum in Geographically Distinct Deep-Sea Hydrothermal Vents. mSystems 2020; 5:e00673-19

8. Rehman ZU, Ali M, Iftikhar H, et al. Genome-resolved metagenomic analysis reveals roles of microbial community members in full-scale seawater reverse osmosis plant. Water Res. 2019; 149:263–271

9. Cuscó A, Pérez D, Viñes J, et al. Long-read metagenomics retrieves complete single-contig bacterial genomes from canine feces. BMC Genomics 2021; 22:330

10. Tett A, Huang KD, Asnicar F, et al. The Prevotella copri Complex Comprises Four Distinct Clades Underrepresented in Westernized Populations. Cell Host Microbe 2019; 26:666-679.e7

11. Bisanz JE, Soto-Perez P, Noecker C, et al. A Genomic Toolkit for the Mechanistic Dissection of Intractable Human Gut Bacteria. Cell Host Microbe 2020; 27:1001-1013.e9

12. Maistrenko OM, Mende DR, Luetge M, et al. Disentangling the impact of environmental and phylogenetic constraints on prokaryotic within-species diversity. ISME J. 2020; 14:1247–1259

13. Zhou Z, Charlesworth J, Achtman M. Accurate reconstruction of bacterial pan- And core genomes with PEPPAN. Genome Res. 2020; 30:1667–1679

14. Park SC, Lee K, Kim YO, et al. Large-scale genomics reveals the genetic characteristics of seven species and importance of phylogenetic distance for estimating pan-genome size. Front. Microbiol. 2019; 10:834

15. Rouli L, Merhej V, Fournier PE, et al. The bacterial pangenome as a new tool for analysing pathogenic bacteria. New Microbes New Infect. 2015; 7:72–85

16. Tettelin H, Riley D, Cattuto C, et al. Comparative genomics: the bacterial pan-genome. Curr. Opin. Microbiol. 2008; 11:472–477

17. Qin X, Galloway-Pẽa JR, Sillanpaa J, et al. Complete genome sequence of Enterococcus faecium strain TX16 and comparative genomic analysis of Enterococcus faecium genomes. BMC Microbiol. 2012; 12:135

18. Chan AP, Sutton G, DePew J, et al. A novel method of consensus pan-chromosome assembly and large-scale comparative analysis reveal the highly flexible pan-genome of Acinetobacter baumannii. Genome Biol. 2015; 16:143

19. Costa SS, Guimarães LC, Silva A, et al. First Steps in the Analysis of Prokaryotic Pan-Genomes. Bioinform. Biol. Insights 2020; 14:1177932220938064
